# Beyond phosphorylation: Putative roles of post-translational modifications in *Plasmodium* sexual stages

**DOI:** 10.1016/j.molbiopara.2021.111406

**Published:** 2021-09

**Authors:** Nila Johnson, Nisha Philip

**Affiliations:** Institute of Immunology and Infection Research, School of Biological Sciences, University of Edinburgh, Edinburgh, EH9 3FL, UK

**Keywords:** Post-translational modifications, Malaria parasite, Plasmodium, Sexual stages

## Abstract

Post-translational modifications (PTMs) allow proteins to regulate their structure, localisation and function in response to cell intrinsic and environmental signals. The diversity and number of modifications on proteins increase the complexity of cellular proteomes by orders of magnitude. Several proteomic and molecular studies have revealed an abundance of PTMs in malaria parasite proteome, where mediators of PTMs play crucial roles in parasite pathogenesis and transmission. In this article, we discuss recent findings in asexual stages of ten diverse PTMs and investigate whether these proteins are expressed in sexual stages. We discovered 25–50 % of proteins exhibiting post-translational modifications in asexual stages are also expressed in sexual stage gametocytes. Moreover we analyse the function of the modified proteins shared with the gametocyte proteome and try to encourage the scientific community to investigate the roles of diverse PTMs beyond phosphorylation in sexual stages which could not only reveal unique aspects of parasite biology, but also uncover new avenues for transmission blocking.

## Highlights

• Extensive proportion of the malaria parasite proteome is post-translationally modified.
• Glycolytic enzymes contain numerous and diverse modifications.
• Cross-talk between PTMs could regulate parasite signalling and metabolic networks.

## 1 Introduction

**Fig. 1:**
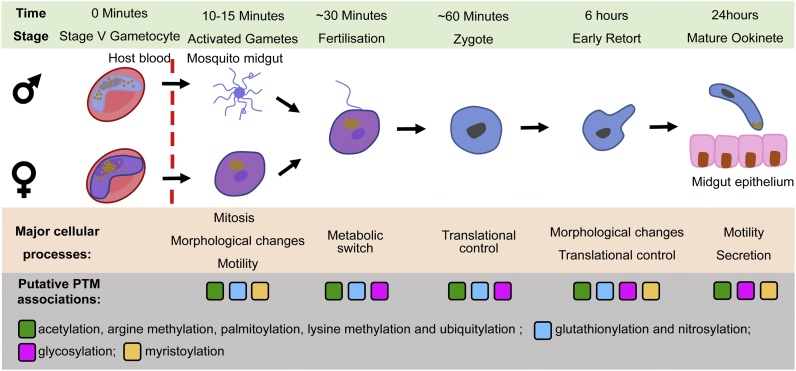
Early transmission stage development of *Plasmodium* and putative PTM involvement. Key cellular processes involved in transmission stage development are listed, along with timing after transfer to the mosquito midgut from the vertebrate host. Quiescent gametocytes are taken up during a blood meal by *Anopheles* mosquitoes where the environmental change induces a developmental switch. Activated gametes round up (*P. falciparum*) and egress (all species) from the erythrocyte, where males replicate their genome 3X and exflagellate into eight motile male microgametes. Male and female gametes fertilise to form an apolar zygote, where temporal release of stored mRNAs for translation is essential to coordinate development into an ookinete. Motile ookinetes invade the midgut epithelium, initiate development into oocysts, which eventually release sporozoites into the mosquito salivary gland ready for transmission back to the mammalian host. In the bottom panel, putative PTMs associated with proteins (minimum of three identified in PTM studies) regulating these key developmental processes are shown.

Malaria maintains a high global disease burden, threatening the health of millions of people. There were ∼229 million cases of malaria worldwide in 2019, resulting in 409,000 deaths [1]. Malaria is caused by apicomplexan parasites of the *Plasmodium* genus, and *Plasmodium falciparum* accounts for the highest global morbidity and mortality [1]. Most cases of malaria can be treated effectively with artemisinin-based combination therapy (ACT), however resistance to ACT is increasing and thus the need for new therapies is growing. Completion of the parasite’s life cycle requires both a vertebrate host (asexual stage) where it causes disease and a mosquito vector (sexual stage) which is essential for parasite transmission. To successfully eradicate malaria, it is essential to develop both curative and transmission blocking strategies. Few approved anti-malarials prevent parasite transmission [2], and to develop new transmission blocking therapeutics we have to improve our understanding of sexual stages. *Plasmodium* parasites are transmitted by the female *Anopheles* mosquito. Upon ingestion during a mosquito bloodmeal, specialised transmission-competent parasites (male and female gametocyte cells) respond to rapid environmental changes in the mosquito gut where tight regulation of the parasite’s DNA replication, protein translation, cell morphological changes, and energy metabolism is essential for the parasite’s life-cycle transition into the vector (Fig. 1).

During host-to-vector transition, the parasite faces the challenge of rapidly responding and adapting to a vastly different and hostile mosquito gut environment. In eukaryotes, response to environmental stimuli are often regulated by post-translational modifications of effector proteins. Not only do PTMs on proteins exponentially expand the functional outputs from a gene, the dynamicity conferred by these modifications permit quick responses to changing environments. The modification of proteins can lead to changes in activity, relocalisation, degradation and recruitment of interacting partners. PTMs can be addition of chemical groups (e.g. phosphorylation, acetylation, methylation, S-nitrosylation), addition of polypeptides (ubiquitylation, SUMOylation), addition of complex groups (glycosylation, prenylation, myristoylation) and proteolytic cleavage. Chemical modification of proteins usually occurs at low stoichiometry creating a significant challenge for PTM identification. Advances in tools for enrichment of specific PTMs and development of powerful mass spectrometry has significantly increased our knowledge of the prevalence and dynamics of PTMs in *Plasmodium*. However a majority of the studies have focussed on the asexual blood stages of the parasite and phosphorylation is the sole PTM of non-histone proteins examined in sexual stage gametocytes [3,4]. Accordingly, beyond phosphorylation, the possible function of PTMs in transmission stage biology is completely unexplored.

Increasing our understanding of PTM regulated protein function in transmission stages will aid the development of transmission blocking drugs. Mediators of PTMs are considered attractive targets for therapeutic intervention for various diseases including malaria [5]. Although protein kinases have been the predominant area of focus, there is emerging interest in disruptors of ubiquitylation [6,7] and myristoylation [8] pathways. Here we examine proteomic studies covering PTMs (excluding phosphorylation) in asexual stages and by comparing to the gametocyte proteome studies, we explore the possible roles for these understudied PTMs in transmission biology. We believe now is an opportune time to identify and mechanistically determine how PTMs beyond phosphorylation regulate malaria parasite transmission.

## 2 Methods

A stringent gametocyte proteomic dataset was generated from three recent proteomic studies [[9], [10], [11]], where any protein present in two out of three studies was included in the final gametocyte proteome list (Supplementary Table 1). Proteomic studies covering PTMs in asexual stages of the human malaria parasite, *Plasmodium falciparum* were collated for the following: acetylation [12,13], glutathionylation [14], glycosylation (O-GlcNAc) [15], methylation [16,17], myristoylation (including GPI anchors) [18], nitrosylation [19], palmitoylation [20], prenylation [21,22], and ubiquitylation [23,24]. The proteins present in these PTM studies were then cross referenced with the gametocyte proteome to produce a list of proteins which could be putatively post-translationally modified in sexual stages. The two acetylation [12,13], prenylation [21,22], and ubiquitylation studies [23,24] were then combined into merged protein lists, where any protein in either study was included (duplicates removed), resulting in lists of 579, 17, and 395 proteins respectively. All proteome lists were produced using R version 4.0.3 [25], package [26], graphs were produced using packages [27]. In order to determine which processes were enriched in this data subset, GO-term analysis of biological process (BP) and molecular function (MF) terms were performed using PlasmoDB 50. Gene Ontology (GO) terms were limited to GO slim terms to limit redundancy and a p value cut-off of 0.01. PlasmoDB GO term enrichments were used to produce Supplementary Fig. 1. GO term enrichment (BP and MF) of the 6 largest PTM datasets (>200 proteins) was performed using the Cytoscape application BINGO [28]; significance level 0.05, multiple testing correction Benjamini & Hochberg False Discovery Rate (FDR). The BINGO networks were then processed in the Cytoscape using Enrichment Map [29] (p value cut off 0.001, q value cut off 0.05, Jaccard coefficient 0.25). GO term clusters were then annotated manually.

## 3 Results and discussion

**Fig. 2:**
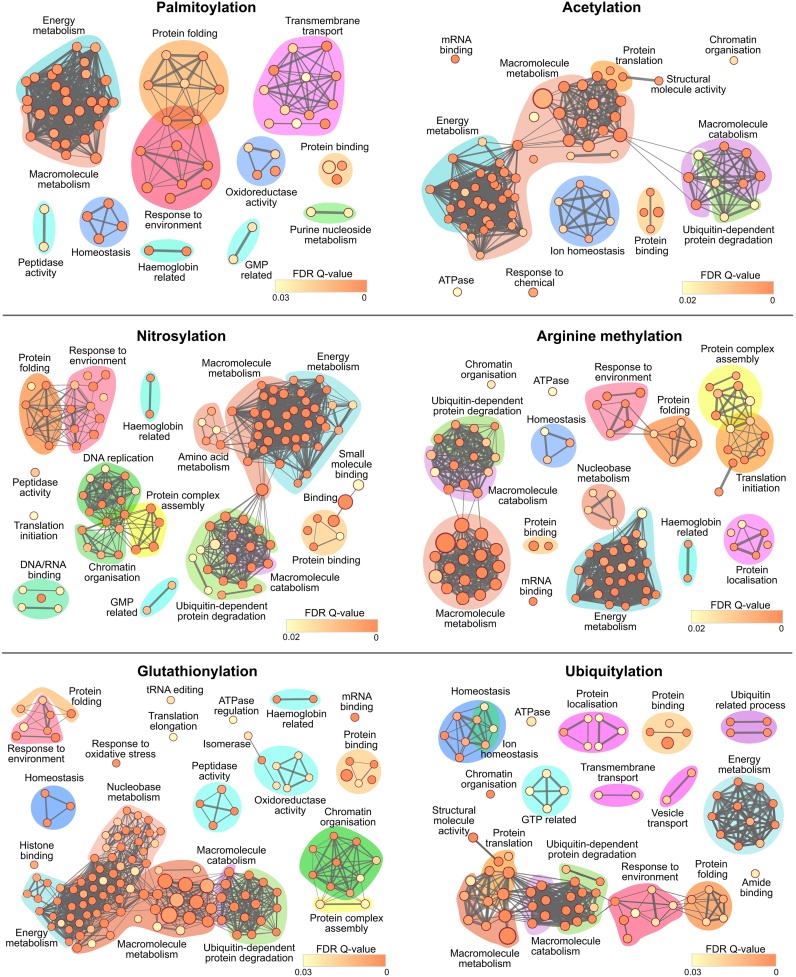
Network maps depicting GO term enrichment of proteins identified from asexual stage PTM studies that are also expressed in gametocytes. Enriched GO terms are represented by nodes, where the colour corresponds to the Q value, and the node size corresponds to the number of proteins found within the term. Edges represent shared proteins between GO terms, where the thickness of the edge represents the relative overlap of proteins. Node sizes and edge thickness are comparable within PTMs but not between. Cluster contents can be found in Supplementary Table 5.

We discovered a significant proportion of proteins identified in PTM studies in the asexual parasites were also detected in the gametocyte proteome suggesting these proteins could be putatively modified in gametocytes and consequently regulate parasite transmission (Table 1 and Supplementary Table 2). While all PTM studies identified putatively modified proteins using mass spectrometry, not everyone identified the site of modification (Table 1). All studies discussed here employed a form of enrichment including direct precipitation of modified proteins/peptides or replacing the PTM with biotin. Acetylation [12,13] and ubiquitylation [23] sites were identified directly from MS spectra. However labile modifications such as nitrosylation or lipid modifications for example prenylation, palmitoylation and myristolylation were replaced and protected by biotin via click chemistry prior to identification by MS. Although biotin switch allowed identification of several modified sites, further technical optimisation is necessary to improve coverage. We have not included histone modifications in our analysis and the reader can refer to studies identifying PTMs in isolated histones [[30], [31], [32]]. These studies discovered extensive acetylation, methylation, ubiquitylation and sumoylation of histones suggesting the importance of histone PTMs for dynamic epigenetic regulation in both asexual and sexual stage biology. In total 1114 proteins showed at least one type of PTM of which 716 proteins exhibited two or more modifications (Supplementary Tables 3 and 5). To assess the functional consequences of the diverse PTMs on proteins identified in the datasets, we first determined the enrichment of GO terms for Molecular Function (MF) and Biological Process (BP) using PlasmoDB (Supplementary Table 4). The 30 % unannotated *Plasmodium* genome can influence GO term analysis. However, the gametocyte proteome dataset examined here has a lower proportion (15 %) of non-curated or non-computed GO terms indicating GO analysis would provide valuable insight into underlying processes regulated by the diverse PTMs and their putative role in sexual stage biology. As expected the number of enriched GO (slim) terms were frequently proportional to size of datasets where smaller dataset (Myristoylation - 28 proteins) generated four GO terms while larger datasets (Acetylation - 579 proteins) generated 26 terms (Supplementary Fig. 1 and Supplementary Table 4). A more detailed GO term enrichment for the six largest PTM datasets were mapped into networks using Cytoscape to determine associations and interconnectedness of various cellular processes (Fig. 2 and Supplementary Table 5).

**Table 1:** Number of proteins identified in each PTM study and also represented in the gametocyte proteome.

All proteins identified in the mentioned studies are collated here with a few adjustments: (i)The Wright et al. study contained both GPI anchored and myristoylated proteins, which the authors distinguished by sensitivity to base treatment. Here the putative GPI and Myristoylated proteins were separately compared against the gametocyte proteome. In addition, only Myristoylated proteins with an N-terminal glycine were included (ii) In the Ponts et al. dataset only rank 1–73 were used, as proteins below rank 73 were identified as low confidence. Text colours denote: Green (modified sites were identified), Orange (some modification sites were identified) and black (proteins were identified as modified, but the site of modification was not determined). The full lists of proteins can be found in Supplementary Table 2.

Although we have attempted to delineate processes related to metabolism, several GO enrichments encompassed in macromolecule and energy metabolism are clearly connected. Further evaluation revealed enrichment of all PTMs except lipidation in carbohydrate metabolism covering glycolytic enzymes. Notably RNA binding was enriched in all chemical modifications including methylation, acetylation and the redox modifications which suggests PTMs could regulate function of RNA binding proteins including the protein translation machinery. Not surprisingly, ubiquitin mediated protein degradation was enriched in the ubiquitome study. Intriguingly, enrichment of ubiquitin mediated degradative processes in all PTM studies except palmitoylation implies that the complex proteasome machinery is exquisitely regulated by several PTMs.

Molecular processes recognised to be important for transmission stage biology include carbohydrate metabolism pathways, DNA replication (male gametogenesis) and RNA metabolism/protein translation. Consequently, we further examined networks associated with metabolic regulation, mitosis and RNA binding proteins. We also discuss proteins which exhibit the highest diversity of PTMs and their possible functions in transmission stages.

### 3.1 Proteins extensively decorated by PTMs

Over 50 % of the proteins identified in the PTM studies exhibited at least two distinct modifications including 118 proteins which were modified by over four different PTMs (Supplementary Table 3 and Supplementary Fig. 2). Up to eight different modifications were observed in a select few proteins including BiP, an ER-chaperone and the glycolytic enzyme, enolase while several other glycolytic enzymes and chaperone proteins are targeted by up to seven PTMs. The high prevalence of chaperones in the PTM studies could be a reflection of protein abundance which results in increased likelihood of detection. On the other hand, diversity in PTMs could allow fine-tuning to maintain specificity of chaperone function. Molecular chaperones such as HSP70 and 90 have a diverse substrate clientele where they can facilitate substrate folding, intracellular transport and assembly of multiprotein complexes [33]. To maintain specificity and accurately target the diverse substrates, activity, localisation and substrate binding of chaperones will require tight regulation. This tight regulation could be enabled by cross-talk between different modifications. In human cells, several PTMs including phosphorylation, acetylation, nitrosylation and methylation impact HSP70 and 90 activity, localisation and substrate binding to main cell homeostasis [34]. Accordingly, the diverse PTMs could regulate *Plasmodium* chaperone function in response to changing environments during parasite transmission. Interestingly a protein phosphatase (PPM2) is decorated by seven distinct PTMs. In *P. berghei*, PPM2 was shown to regulate sex allocation where in the absence of the phosphatase, not only was gametocyte production reduced, the male:female ratio was altered from 1:3 in wild type to 1:1 in the mutant [35]. This leads to an intriguing possibility where PTMs could regulate PPM2 function to impact the parasite’s gender allocation.

### 3.2 Metabolic regulation

Upon transmission from the vertebrate host bloodstream to the mosquito midgut, *Plasmodium* parasites experience a vastly different environment where parasites dramatically rewire their metabolism. While asexual stage parasites primarily utilise glycolysis for their energy needs, in the mosquito environment, they become increasingly reliant on the tricarboxylic acid (TCA) metabolism [36]. Although male gametogenesis can be fuelled by both glycolysis and the TCA cycle, the parasite is only critically dependent on glycolysis for the gamete’s flagellar motility [37]. However female cells and post-fertilisation development stages switch more heavily to a reliance on the glutamine fed TCA cycle [38,39].

Energy metabolism and macromolecule biosynthesis were enriched in all PTM datasets except for myristolyation and prenylation (Fig. 2, Supplementary Fig. 1 and Supplementary Table 4). The groups predominantly contained glycolytic enzymes including hexokinase, glyceraldehyde 3 phosphate dehydrogenase (GAPDH), phosphoglycerate kinase, phosphoglycerate mutase, enolase, pyruvate kinase (PK) and lactate dehydrogenase. Emerging evidence suggests that cancer cells reprogram their metabolism by post-translationally modifying glycolytic enzymes [40]. Accordingly, diverse PTMs control the activity, localisation and stability of PK, where acetylation targets the protein for lysosomal degradation, while methylation and glycosylation re-localise the enzyme from the cytosol to mitochondria and nucleus respectively. In *P. falciparum* S-glutathionylation reversibly inhibits GAPDH and PK activity, demonstrating regulatory functions for protein modifications during glycolysis [14]. All the above seven glycolytic enzymes show at least five types of PTMs, suggesting cross-talk between several PTMs may allow fine-tuned regulation of the glycolytic pathway and could additionally play a role in metabolic re-wiring upon parasite transmission.

Metabolic plasticity in asexual blood stages allows overcoming the requirement of the TCA cycle for energy production. However, a functional TCA cycle is essential for adaptation to the mosquito environment. Gene deletion studies have identified requirement for aconitase during male gametocytogenesis (*P. falciparum*) and gametogenesis (*P. berghei*), while α-ketoglutarate dehydrogenase (KDH) is essential for oocyst formation [36,38]. Six of the eight mitochondrial TCA enzymes are post-translationally modified where both acotinase and KDH are modified by palmitoylation suggesting a regulatory role on these essential enzymes.

Taken together our analyses suggest key functions for numerous PTMs in metabolic regulation of the parasite in asexual stages. The expression and subsequent function of these enzymes in transmission stage biology suggests diverse PTMs could interact and fine tune the parasite’s metabolic choices as it adapts to the vector environment.

### 3.3 DNA binding and replication

Rapid DNA replication is a hallmark of male gamete formation where the male genome undergoes three rounds of replication interspersed with three rounds of endomitosis within eight minutes of sensing the mosquito environment [41,42]. Successfully coordinating this complex process in a limited timeframe is a challenging task requiring exquisite control of DNA replication. We found DNA binding proteins such as proliferating cell nuclear antigen 1 (PCNA1) are heavily modified by acetylation, methylation, glutathionylation, nitrosylation palmitoylation and ubiquitylation. PCNA endows polymerases with the high processivity required for duplicating entire genomes [43], a process essential for male gamete formation. Male gamete formation will also require nucleosome assembly post DNA replication. We discovered that chromatin assembly factor 1 subunit C [44,45], a protein implicated in depositing histones on newly replicated DNA is modified by redox modifications (glutathionylation, nitrosylation) and acetylation. Moreover, DNA binding or/and chromatin assembly proteins were enriched in the arginine methylation, acetylation, nitrosylation, and ubiquitylation datasets. All four studies contained minichromosome maintenance (MCM) DNA replication factors exhibiting arginine methylation (MCM4/5/6/7) acetylation (MCM3/4/6), nitrosylation (MCM2/3/4/5/6/7) and ubiquitylation (MCM2/7). The MCM complex initiates DNA replication, and has been implicated in early male gamete DNA replication through its association with and phosphorylation by CDPK4 [3]. The PTM studies indicate MCM may be additionally regulated by interplay of multiple modifications. Furthermore, modifications on subunits of DNA polymerase, replication factor C, DNA ligase I, DNA topoisomerase II, and ORC subunit 1 suggests acetylation could control temporal regulation of DNA replication in male gametes. Taken together the analysis suggests that the atypical cell cycle driving male gamete formation could predominantly be regulated by cross-talk between phosphorylation and acetylation.

### 3.4 RNA binding proteins and translation

Post-transcriptional regulation is critical to the malaria parasite’s sophisticated developmental programs where RNA binding proteins (RBP) control maturation, localisation, decoding and stability of mRNAs. In our analysis of the published PTM datasets, we found ‘RNA binding’ and ‘Translation’ enriched in 80 % of the studies, where the identified proteins were predicted to be involved in splicing, ribosome integrity, RNA decay and translation regulation.

Bioinformatic studies of the *Plasmodium* genome have identified over 180 RBPs, where 42 members are predicted to function in mRNA splicing [46]. The mRNA spliceosome assembly has already been associated with PTM regulation, namely arginine methylation [47]. In addition to methylation, members of the spliceosome complex were also identified in acetylation, glutathionylation, palmitoylation, nitrosylation, and ubiquitylation datasets implying the importance of diverse PTMs in parasite spliceosome function.

RNA granules are higher order assemblies of RNA and proteins, which can regulate timing of mRNA translation. In the malaria parasite, translational repression and temporal activation of protein synthesis are well recognised processes that critically regulate successful host↔vector transition [48,49]. The female gametocyte holds a significant portion of its transcriptome in RNA storage granules in a translationally repressed state. Only upon encountering the mosquito environment, the transcripts are temporally licensed for translation. In *P. berghei* gametocytes, the RNA granules contain several RBPs including the RNA helicase DOZI, Alba1-4, Bruno homolog, Sm-like CITH and Poly A binding proteins where DOZI and CITH are known to maintain mRNA stability [50]. We found PABP1 and Alba 4 contain at least six types of PTMs including acetylation, ubiquitylation, both arginine and lysine methylation, glutathionylation, nitrosylation or palmitoylation. Moreover DOZI, Musashi, Alba1-3, PABP 2/3 and CITH contained between one to five different modifications. It is currently unknown how temporal licencing of translation of mRNA from RNA granules is achieved. Considering the abundance of PTMs in RNA granule proteins, the co-ordination of temporal waves of translation could be achieved by dynamic and diverse modification of RBPs.

In addition to RBPs, several components of the translational machinery including ribosomal subunits, initiation and elongation factors (eIF and eEF) are modified by a combination of several PTMs including acetylation, glutathionylation, glycosylation, nitrosylation, methylation (Arginine and Lysine), palmitoylation or ubiquitylation. Elongation factor 2 (eEF-2) and the ribosomal stalk protein P0 were among the most diversely modified proteins displaying seven of the above eight modifications. Interestingly a specific inhibitor of *Plasmodium* eEF-2 (M5717) is in preclinical development as a promising multi-stage antimalarial [51]. The diversity of PTMs on eEF-2 suggest not only a complex interplay of regulation, but also the exciting possibility that mediators of eEF-2 modification could be powerful targets for antimalarial therapy including transmission.

## 4 Conclusions and the future

Our analyses indicate that diverse PTMs could orchestrate spatial and temporal control of molecular processes essential for parasite transmission. It is important to note that the gametocyte specific proteome (231 proteins) is absent from our analyses. Although our study suggests several post-translationally modified proteins could regulate parasite transmission, only a gametocyte specific PTM analyses would confidently identify pathways which are significant and also unique to gametocyte biology. Importantly, we found that gametocytes express several writers and erasers of the PTMs discussed in our study, strongly suggesting dynamic protein modifications could coordinate parasite response and adaption to the mosquito environment (Supplementary Table 6). Expression of seven acetyl, eleven methyl and three palmitoyl transferases in the gametocyte proteome suggest PTMs including acetylation, methylation and palmitoylation can regulate *Plasmodium* transmission. Indeed, small molecules targeting lysine acetylation and methylation exhibit activity against gametocytes and gametes [52,53]. Moreover, both chemical and genetic tools have demonstrated the requirement for palmitoyl-S-acyl-transferase (DHHC2 and 10) for ookinete development and malaria transmission [[54], [55], [56]]. Components of ubiquitin machinery including ubiquitin ligases (E2s and E3s) and deubiquitinases are expressed in gametocytes where gene deletions in several enzymes prevent parasite transmission, highlighting the importance of this key eukaryotic PTM during sexual stage biology [57].

While the primary goal of this study was to investigate whether proteins post-translationally modified in asexual stages could also regulate transmission stages, it became increasingly clear that occurrence of diverse PTMs on individual proteins is common. Co-existence of several PTMs on the same protein molecule or coordination of different PTMs if occurring exclusively, could not only fine-tune, but also expand function of signalling pathways in both asexual stages and sexual stages. Delineating complex signalling networks facilitated by cross-talk between diverse PTMs will be a future challenge. Development of powerful computational and experimental tools will be vital to decipher the organisation and biological outcomes of the signalling pathways. With the advent of CRISPR-Cas9 gene editing and successful application to *Plasmodium* parasites, the ability to dissect roles of specific amino acids targeted by PTMs will increasingly improve our understanding of protein regulation. Directly targeting the modified proteins or the responsible PTM machinery could vastly increase our repertoire of targets for both curative and transmission blocking strategies.

## CRediT authorship contribution statement

**Nila Johnson:** Methodology, Validation, Investigation and Writing. **Nisha Philip:** Supervision, Methodology, Validation, Investigation and Writing.
