# Supplementary material for: Beyond phosphorylation: Putative roles of post-translational modifications in Plasmodium sexual stages
Source: Mol Biochem Parasitol. 2021 Sep;245:111406. doi: 10.1016/j.molbiopara.2021.111406 (PMC8505795; doi:10.1016/j.molbiopara.2021.111406)
Supplement: Supplementary file 1 [file mmc1.docx]

**Supplementary figure legends**

Supplementary Figure 1: GO term enrichment of proteins identified from asexual stage studies that are also expressed in gametocytes.

GO-term analysis of biological process (BP) and molecular function (MF) terms were performed using PlasmoDB 50. Gene Ontology (GO) terms were limited to GO slim terms to limit redundancy and a p value cut-off of 0.01. GO term enrichments are visualised using R (ggplot 2) as bubble plots. Bubble size represents the number of proteins in that GO term. FDR adjusted Q value reported is the Benjamini & Hochberg FDR Q value calculated by PlasmoDB (only displaying terms with Q < 0.05). The terms “biological process” and “molecular function” were removed from the graph to aid visualisation. Bubble size is scaled equally across all PTMs.

Supplementary Figure 2: Overlap of the six largest PTM datasets

The graph denotes the degree of overlap in proteins identified in the six largest PTM datasets to visualise interplay between PTMs. The total number of proteins identified for each modification is depicted in bar charts on the left. The panel at the base of the main graph displays the thirty top unique and overlapping modifications.
