# Supplementary material for: Beyond phosphorylation: Putative roles of post-translational modifications in Plasmodium sexual stages
Source: Mol Biochem Parasitol. 2021 Sep;245:111406. doi: 10.1016/j.molbiopara.2021.111406 (PMC8505795; doi:10.1016/j.molbiopara.2021.111406)
Supplement: Supplementary file 2 [file mmc2.pdf]

Supplementary Figure 1

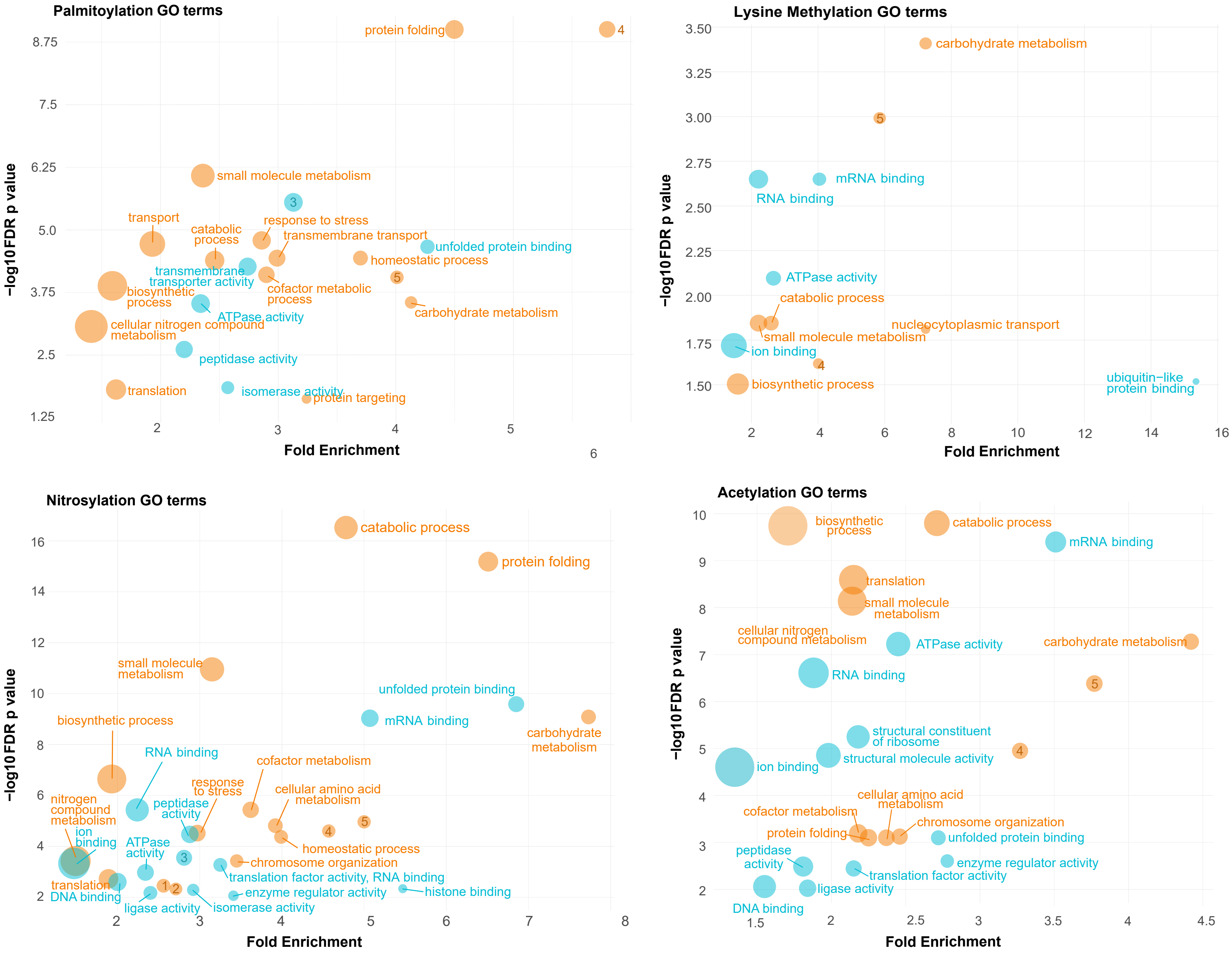

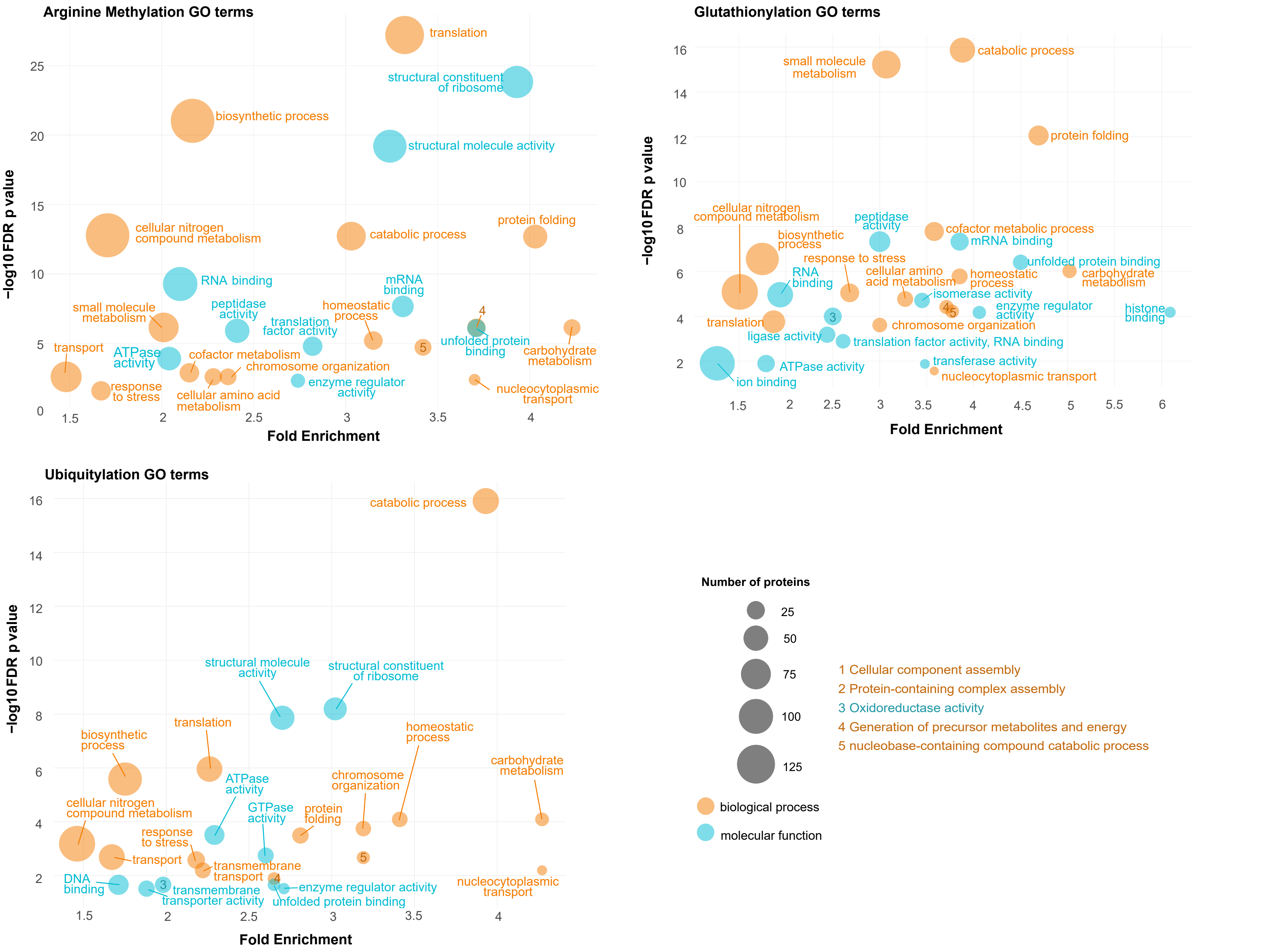

Arginine Methylation GO terms
translation
structural constituent of ribosome
biosynthetic process
structural molecule activity
cellular nitrogen compound metabolism
catabolic process
protein folding
RNA binding
mRNA binding
small molecule metabolism
peptidase activity
homeostatic process
translation factor activity
unfolded protein binding
carbohydrate metabolism
transport
ATPase activity
cofactor metabolism
chromosome organization
enzyme regulator activity
response to stress
cellular amino acid metabolism
nucleocytoplasmic transport
−log10 FDR p value
Fold Enrichment
Glutathionylation GO terms
catabolic process
small molecule metabolism
protein folding
cellular nitrogen compound metabolism
peptidase activity
cofactor metabolic process
biosynthetic process
mRNA binding
response to stress
unfolded protein binding
RNA binding
cellular amino acid metabolism
homeostatic process
carbohydrate metabolism
isomerase activity
enzyme regulator activity
histone binding
translation
chromosome organization
ligase activity
translation factor activity, RNA binding
transferase activity
ATPase activity
nucleocytoplasmic transport
ion binding
Ubiquitylation GO terms
catabolic process
structural molecule activity
structural constituent of ribosome
translation
homeostatic process
biosynthetic process
carbohydrate metabolism
ATPase activity
chromosome organization
cellular nitrogen compound metabolism
GTPase activity
protein folding
response to stress
transport
transmembrane transport
DNA binding
transmembrane transporter activity
enzyme regulator activity
unfolded protein binding
nucleocytoplasmic transport
Number of proteins
25
50
75
100
125
biological process
molecular function
1 Cellular component assembly
2 Protein-containing complex assembly
3 Oxidoreductase activity
4 Generation of precursor metabolites and energy
5 nucleobase-containing compound catabolic process
