# Supplementary figures and images for: Beyond phosphorylation: Putative roles of post-translational modifications in Plasmodium sexual stages
Source: Mol Biochem Parasitol. 2021 Sep;245:111406. doi: 10.1016/j.molbiopara.2021.111406 (PMC8505795; doi:10.1016/j.molbiopara.2021.111406)

Supplementary Figure 2

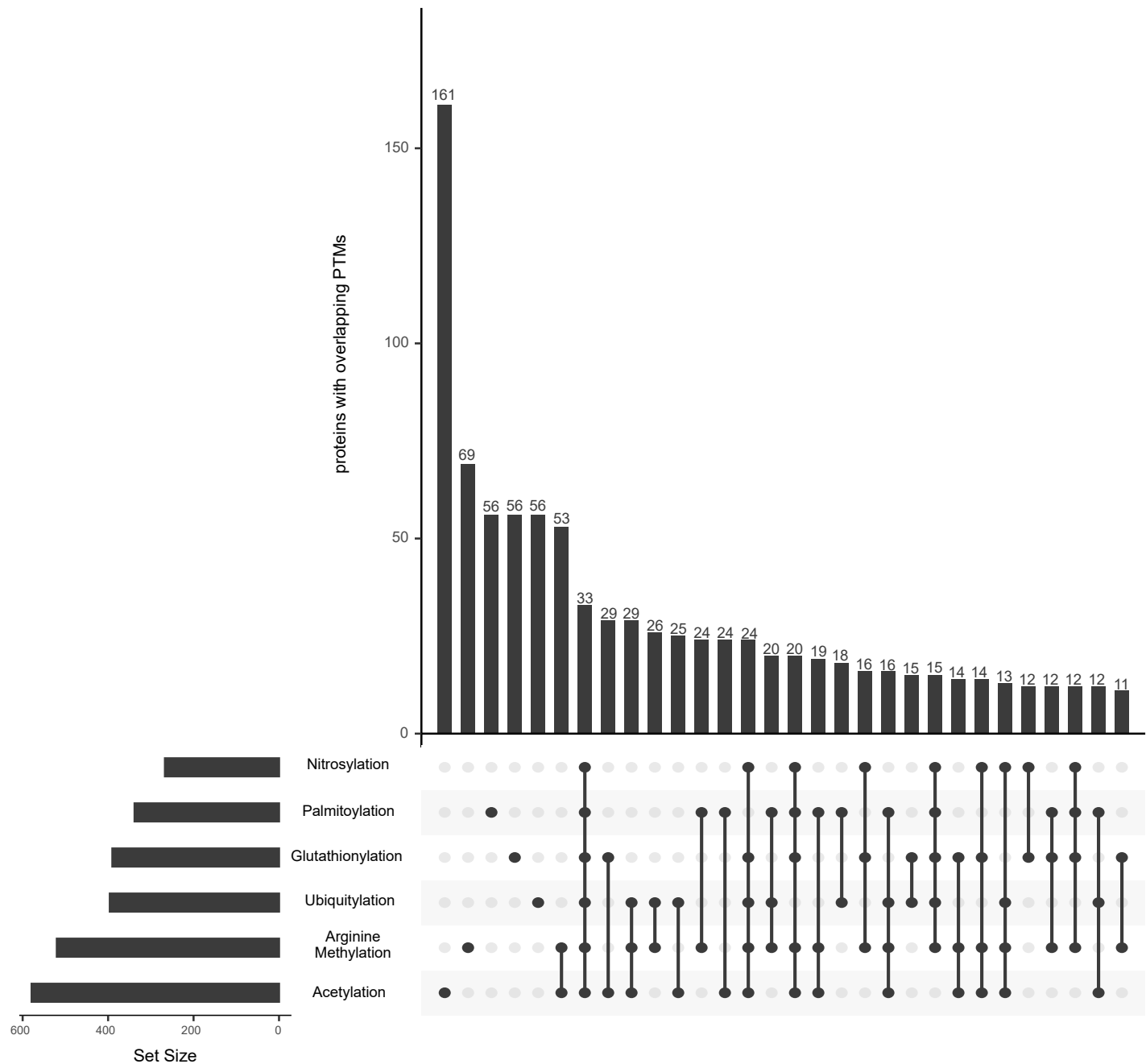

Supplement: Supplementary file 3 [file mmc3.pdf]
