# Supplementary material for: Beyond phosphorylation: Putative roles of post-translational modifications in Plasmodium sexual stages
Source: Mol Biochem Parasitol. 2021 Sep;245:111406. doi: 10.1016/j.molbiopara.2021.111406 (PMC8505795; doi:10.1016/j.molbiopara.2021.111406)
Supplement: Supplementary file 9 [file mmc9.docx]

| Supplementary Table 6: PTM descriptions and PTM mediating machinery identified in the gametocyte proteome | | |
| --- | --- | --- |
| PTM | Description | Mediators in the gametocyte proteome |
| Acetylation | Acetylation is the addition of an acetyl group to a target protein at the either N-terminus or an internal Lysine. Acetylation is associated with changes in gene expression (histone acetylation), protein localisation, protein activation, and promoting protein-protein interactions. | histone acetyltransferase GCN5 [PF3D7_0823300](https://plasmodb.org/plasmo/app/record/gene/PF3D7_0823300)  N-alpha-acetyltransferase 15, NatA auxiliary subunit, putative [PF3D7_0303700](https://plasmodb.org/plasmo/app/record/gene/PF3D7_0303700)  N-acetyltransferase, GNAT family, putative [PF3D7_1437000](https://plasmodb.org/plasmo/app/record/gene/PF3D7_1437000)  [PF3D7_0303700](https://plasmodb.org/plasmo/app/record/gene/PF3D7_0303700) lipoamide acyltransferase  N-acetyltransferase, GNAT family, putative [PF3D7_1020700](https://plasmodb.org/plasmo/app/record/gene/PF3D7_1020700)  phospholipid or glycerol acyltransferase, putative [PF3D7_0914200](https://plasmodb.org/plasmo/app/record/gene/PF3D7_0914200)  histone deacetylase, putative [PF3D7_1472200](https://plasmodb.org/plasmo/app/record/gene/PF3D7_1472200) |
| Glutathionylation | (S-)Glutathionylation is the addition of a Glutathione (tripeptide: Glycine, Cysteine, Glutamic acid) to a Cysteine on the target protein, via disulphide bonds between Cysteines. Glutathionylation is associated with the response to oxidative stress, and is known to modulate signalling pathways via enzyme activation/deactivation. | glutaredoxin 1 [PF3D7_0306300](https://plasmodb.org/plasmo/app/record/gene/PF3D7_0306300)  glutaredoxin-like protein [PF3D7_0606900](https://plasmodb.org/plasmo/app/record/gene/PF3D7_0606900) |
| Glycosylation | Glycosylation attaches a carbohydrate molecule to a protein target. N-linked glycosylation adds a carbohydrate to the nitrogen of an asparagine or arginine side chain. O-linked glycosylation adds a carbohydrate to the oxygen of a Serine, Threonine, Tyrosine, hydroxylysine or hydroxyproline side chains. Glycosylation is involved in protein folding, cell attachment, and enzyme activation/deactivation. | dolichyl-phosphate-mannose--protein mannosyltransferase [PF3D7_1010700](https://plasmodb.org/plasmo/app/record/gene/PF3D7_1010700)  Dpy-19-like C-mannosyltransferase [PF3D7_0806200](https://plasmodb.org/plasmo/app/record/gene/PF3D7_0806200)  Dolichol-phosphate mannosyltransferase polypeptide 1 (DPM1) PF3D7_1141600  UTP-glucose-1-phosphate uridylyltransferase (UGP) or UDP-sugar pyrophosphorylase (USP) PF3D7_0517500  Phosphoglucomutase (PGM) PF3D7_1120100 |
| Methylation | Methylation is the addition of a methyl (CH_3_) group to a target protein via nitrogen containing Lysine or Arginine resides. Proteins can also be methylated at the N-terminus via the amino group. Methylation is typically associated with epigenetic control via histones, but is also involved in protein-protein interactions and other functions on non-ribosomal proteins. | 2-methoxy-6-polyprenyl-1,4-benzoquinol methylase, mitochondrial [PF3D7_0204900](https://plasmodb.org/plasmo/app/record/gene/PF3D7_0204900)  methyltransferase, putative [PF3D7_0422900](https://plasmodb.org/plasmo/app/record/gene/PF3D7_0422900)  histone-arginine methyltransferase CARM1, putative [PF3D7_0811500](https://plasmodb.org/plasmo/app/record/gene/PF3D7_0811500)  methyltransferase, putative [PF3D7_1303100](https://plasmodb.org/plasmo/app/record/gene/PF3D7_1303100)  phosphoethanolamine N-methyltransferase [PF3D7_1343000](https://plasmodb.org/plasmo/app/record/gene/PF3D7_1343000)  protein arginine N-methyltransferase 5, putative [PF3D7_1361000](https://plasmodb.org/plasmo/app/record/gene/PF3D7_1361000) multifunctional methyltransferase subunit TRM112, putative [PF3D7_1407500](https://plasmodb.org/plasmo/app/record/gene/PF3D7_1407500)  protein arginine N-methyltransferase 1 [PF3D7_1426200](https://plasmodb.org/plasmo/app/record/gene/PF3D7_1426200)  protein-L-isoaspartate(D-aspartate) O-methyltransferase, putative [PF3D7_1432700](https://plasmodb.org/plasmo/app/record/gene/PF3D7_1432700)  leucine carboxyl methyltransferase, putative [PF3D7_1439700](https://plasmodb.org/plasmo/app/record/gene/PF3D7_1439700)  methyltransferase, putative [PF3D7_1455200](https://plasmodb.org/plasmo/app/record/gene/PF3D7_1455200) |
| Nitrosylation | Protein (S-)Nitrosylation involves the addition of a nitric oxide (NO) group to a Cysteine thiol within the target protein. Nitrosylation is involved in intracellular signal transduction. | Thioredoxin 1 PF3D7_1457200 |
| Palmitoylation | Palmitoylation is the addition of fatty acid groups such as palmitic acid to a Cysteine (S-palmitoylation), or Serine/Threonine (O-palmitoylation) residue on a target protein. Palmitoylation is associated with increased hydrophobicity of the target, and promotes interaction with membranes. Palmitoylation is thought to play a role in intracellular trafficking and promoting protein-protein interactions. | palmitoyltransferase DHHC1 PF3D7_0303400  palmitoyltransferase DHHC2 PF3D7_0609800  palmitoyltransferase DHHC5 PF3D7_1322500 |
| Prenylation | Protein prenylation is the addition of a farnesyl or geranylgeranyl molecule to the C-terminal Cysteine of a target protein. Prenylation facilitates protein attachment to cell membranes, and can promote protein-protein interactions. | bifunctional farnesyl/geranylgeranyl diphosphate synthase PF3D7_1128400 |
| Ubiquitylation | Ubiquitylation is the addition of one or multiple (chained) ubiquitin (small protein) moieties to a target protein at a Lysine, Cysteine, Serine, or Threonine residue, or the amino group of the N-terminus. Ubiquitylation has a range of effects including protein degradation, relocalisation, activation/deactivation, and interactions. | HECT-type E3 ubiquitin ligase UT [PF3D7_0704600](https://plasmodb.org/plasmo/app/record/gene/PF3D7_0704600)  HECT-like E3 ubiquitin ligase, putative [PF3D7_0826100](https://plasmodb.org/plasmo/app/record/gene/PF3D7_0826100)  ubiquitin conjugation factor E4 B, putative [PF3D7_0826500](https://plasmodb.org/plasmo/app/record/gene/PF3D7_0826500)  ubiquitin carboxyl-terminal hydrolase isozyme L3 [PF3D7_1460400](https://plasmodb.org/plasmo/app/record/gene/PF3D7_1460400)  polyubiquitin [PF3D7_1211800](https://plasmodb.org/plasmo/app/record/gene/PF3D7_1211800)  ubiquitin-conjugating enzyme E2, putative [PF3D7_0305700](https://plasmodb.org/plasmo/app/record/gene/PF3D7_0305700)  ubiquitin-conjugating enzyme E2 N, putative [PF3D7_0527100](https://plasmodb.org/plasmo/app/record/gene/PF3D7_0527100)  ubiquitin-conjugating enzyme E2 PEX4, putative [PF3D7_0606200](https://plasmodb.org/plasmo/app/record/gene/PF3D7_0606200)  ubiquitin-conjugating enzyme E2, putative [PF3D7_0812600](https://plasmodb.org/plasmo/app/record/gene/PF3D7_0812600)  ubiquitin-conjugating enzyme E2, putative [PF3D7_0921000](https://plasmodb.org/plasmo/app/record/gene/PF3D7_0921000) ubiquitin-conjugating enzyme E2, putative [PF3D7_1033900](https://plasmodb.org/plasmo/app/record/gene/PF3D7_1033900)  ubiquitin-conjugating enzyme E2 [PF3D7_1203900](https://plasmodb.org/plasmo/app/record/gene/PF3D7_1203900)  ubiquitin-conjugating enzyme E2, putative [PF3D7_1356300](https://plasmodb.org/plasmo/app/record/gene/PF3D7_1356300)  HECT-domain (ubiquitin-transferase), putative [PF3D7_0628100](https://plasmodb.org/plasmo/app/record/gene/PF3D7_0628100)  ubiquitin carboxyl-terminal hydrolase 2, putative [PF3D7_0516700](https://plasmodb.org/plasmo/app/record/gene/PF3D7_0516700)  ubiquitin carboxyl-terminal hydrolase, putative [PF3D7_0726500](https://plasmodb.org/plasmo/app/record/gene/PF3D7_0726500)  ubiquitin carboxyl-terminal hydrolase 13, putative [PF3D7_0413900](https://plasmodb.org/plasmo/app/record/gene/PF3D7_0413900)  ubiquitin specific protease, putative [PF3D7_0904600](https://plasmodb.org/plasmo/app/record/gene/PF3D7_0904600)  ubiquitin carboxyl-terminal hydrolase isozyme L3 [PF3D7_1460400](https://plasmodb.org/plasmo/app/record/gene/PF3D7_1460400)  ubiquitin carboxyl-terminal hydrolase UCH54 [PF3D7_1117100](https://plasmodb.org/plasmo/app/record/gene/PF3D7_1117100)  ubiquitin carboxyl-terminal hydrolase 14 [PF3D7_0527200](https://plasmodb.org/plasmo/app/record/gene/PF3D7_0527200)  ataxin-3, putative[PF3D7_1226800](https://plasmodb.org/plasmo/app/record/gene/PF3D7_1226800)  26S proteasome regulatory subunit RPN11, putative [PF3D7_1368100](https://plasmodb.org/plasmo/app/record/gene/PF3D7_1368100)  26S proteasome regulatory subunit RPN8, putative [PF3D7_0912900](https://plasmodb.org/plasmo/app/record/gene/PF3D7_0912900)  eukaryotic translation initiation factor 3 subunit F, putative [PF3D7_0918300](https://plasmodb.org/plasmo/app/record/gene/PF3D7_0918300)  pre-mRNA-processing-splicing factor 8, putative [PF3D7_0405400](https://plasmodb.org/plasmo/app/record/gene/PF3D7_0405400)  ER membrane protein complex subunit 8, putative [PF3D7_1139900](https://plasmodb.org/plasmo/app/record/gene/PF3D7_1139900)  PPPDE peptidase domain-containing protein, putative [PF3D7_1217900](https://plasmodb.org/plasmo/app/record/gene/PF3D7_1217900)  OTU domain-containing protein, putative [PF3D7_1141700](https://plasmodb.org/plasmo/app/record/gene/PF3D7_1141700) |
